# Assessment of physicians’ addressing sexuality in elderly patients with chronic pain

**DOI:** 10.1590/S1679-45082016AO3556

**Published:** 2016

**Authors:** Guilherme Liausu Cherpak, Fânia Cristina dos Santos

**Affiliations:** 1 Universidade Federal de São Paulo, São Paulo, SP, Brazil.

**Keywords:** Sexuality, Aged, Chronic pain, Practice patterns, physicians’, Education, medical

## Abstract

**Objective:**

To determine the frequency with which physicians address their older adult patients with chronic pain about the issue of sexuality.

**Methods:**

It is a cross sectional, descriptive, analytical study in which physicians answered a questionnaire comprising questions related to addressing the issue of sexuality during appointments.

**Results:**

A sample of 155 physicians was obtained, 63.9% stated they did not address sexuality in medical interviews and 23.2% did it most of the time. The main reasons for not addressing were lack of time, fear of embarrassing the patient and technical inability to address the issue.

**Conclusion:**

There is a need to develop strategies to increase and improve addressing of sexuality in elderly patients with chronic pain, in order to have better quality of life.

## RESUMO

**Objetivo:**

Determinar a frequência com que os médicos abordam o assunto de sexualidade com seus pacientes mais idosos com dor crônica.

**Métodos:**

Este é um estudo transversal, descritivo, analítico em que os médicos responderam a um questionário com perguntas relativas à abordagem de sexualidade nas consultas.

**Resultados:**

Uma amostra de 155 médicos foi obtida, 63,9% afirmaram que não realizavam a abordagem da sexualidade e 23,2% afirmaram fazê-la na maior parte das vezes. As principais razões para não abordarem foram falta de tempo, medo de constranger o paciente e incapacidade técnica para abordar o assunto.

**Conclusão:**

Existe uma necessidade de desenvolver estratégias para aumentar e melhorar a abordagem de sexualidade em idosos com dor crônica, de forma a melhorar sua qualidade de vida.

## INTRODUCTION

There has been a significant shift in the population design in the world. The number of elderly in the total population is growing, especially in developing countries, like Brazil.^(1,2)^ Quality of life awareness and public health strategies are required to ensure that these older adults continue to have good life conditions.

Older adults usually neglect their sexuality.^(2-4)^ This happens as a result of three main incorrect concepts: First, sexuality in the elderly does not exist. It is known that older adults have sexual activity. According to Helgason et al.,^(5)^ 46% of men aged 70-80 years, in the Swedish population, reported having at least one orgasm in the previous month. In the previous year, according to the study conducted by Lindau et al.,^(2)^ 60% of elderly men and 30% of elderly women had had sexual intercourse. Second: sexuality in the elderly is wrong. Many elders are embarrassed to maintain or discuss their sex life because of stereotypical images of asexual elders,^(6)^ while, according to Drench et al.,^(7)^ elderly people are afraid of being perceived as perverted or luxurious for having an active sex life. Third: sexuality in the elderly is funny. According to Bytheway et al.,^(8)^ sexual difficulties in the elderly are subject to humor.

Sexuality in the elderly is related to better cardiovascular health,^(9)^ quality of life and mood,^(10)^ and longevity.^(11)^ There are, however, certain dysfunctions that appear at that age that impose barriers to continuing a good sexual life. In addition to the physiological changes, such as diminished lubrication and slower sexual response,^(12)^ there is the problem of multiple morbidity^(13)^ and social difficulties (*e.g.,* lack of a partner, lack of privacy).^(14)^

Additionally, regarding previously described barriers, chronic pain is an important factor to complicate a rather difficult sexual activity. According to the International Association for the Study of Pain,^(15)^ pain is an unpleasant sensory and emotional experience, associated with actual or potential tissue damage. It becomes chronic when it surpasses the regular healing time and involves emotional aspects and central sensitization. Chronic pain is a prevalent condition in the elderly. Around the world, it varies from 48 to 83% of older adults,^(16,17)^ while, in Brazil, it is 51.44%.^(18)^ Patients with chronic pain also suffer from sexual dysfunctions. According to Ambler et al.,^(19)^ 73% of chronic pain patients reported sexual dysfunctions, while Bahouq et al.^(20)^ found that 81% of chronic back pain patients reported sexual problems.

A major problem is that patients with sexual dysfunctions do not discuss the issue with their physicians. Gott et al.^(21)^ found that only 4-6% of patients sought medical assistance, and the main causes were the sociodemographic characteristics of the physician, the patient’s idea about sexuality in the elderly, shame due to the fact they think it is related to normal aging, and unawareness of the existence of specialized services. In a worldwide study,^(22)^ 9% of patients with sexual dysfunctions sought assistance in the previous year, and, in another study^(2)^ representative of the American population, 38% of men and 22% of women with sexual dysfunctions sought medical care.

In a qualitative study,^(23)^ the main barriers to communication about sexuality between elderly women and physicians appeared related to the patient or physician. The patient-related barriers were intimidation, shame, fear of disrespecting the physician, sociodemographic differences, fear of exposing sexual orientation, and fear of inability or lack of interest by the physician in the problem.

The physician-related barriers were the belief that senior people are not interested in sex, perceiving the patient as similar to the physician’s own parent, inability to address the issue, the belief that another type of doctor is responsible for that issue, worrying more about diseases than quality of life and poor quality of life prevention, lack of time, religious or his/her own sexual issues, and feeling embarrassed in dealing with the sexuality of older ladies.^(23)^

Thus, chronic pain interferes in a healthy sex life, and elderly patients dealing with these problems are unable to discuss the issue with their physicians. The main objective of this study is to determine the prevalence of addressing the issue of sexuality in medical interviews of elderly patients, as well as the factors related to not addressing the issue with the individual. A better understanding of this lack of addressing the issue can help develop teaching strategies to change the medical educational paradigm, promoting better health care for the elderly population.

## OBJECTIVE

To determine the frequency with which physicians address their older adult patients with chronic pain about the issue of sexuality.

## METHODS

This was a cross-sectional, observational, descriptive-analytical study performed at a teaching hospital, located in the city of São Paulo, in the Southeast of Brazil, with several physician specialties who provide care to elderly patients with chronic pain.

The sample size calculation resulted in 119 participants; however, an actual sample of 155 physicians was obtained.

To calculate the sample size, the following equation^(24)^ was used:


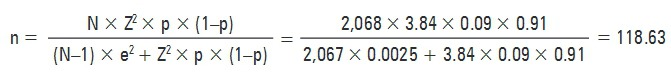


In this equation, **n** is the sample size and **N** is the universe size (here, the number of physicians working in the referred specialties in the hospital was 2,068). **Z** is a constant of the critical value to obtain the desired confidence level. For 95%, **Z** is 1.96. The variable **e** is the maximum error margin accepted-in this case, 5%-and **p** is the literature-based prevalence - in this case, 9%.

There was no restriction on their specialties, years of experience practicing medicine, or position in the hospital (resident or attending physician), except those specialties that naturally address the issue of sexuality as part of their routine, such as urology and gynecology. The reason is that we wished to study the physicians who provide care to elderly with chronic pain, but who do not focus on sexuality frequently in their routines.

A 21-question self-administered closed-question survey relating to clinical practice was given to physicians who practiced specialties that usually involve examining elderly patients. The surveys were distributed individually at clinical meetings, clinics, and at the Hospital of the *Universidade Federal de São Paulo*. To assure the participants’ anonymity, each questionnaire was detached From the Consent Terms and placed in an opaque envelope for separate analysis. The questions were created by the authors and were based on previous qualitative and quantitative studies related to the topic and were all multiple-choice questions.

The participants were asked about their specialty, years of experience, mean age of their elderly patients, if the patients had chronic pain and what type of pain, if they had any training in addressing sexuality, and if they perceived sex and sexuality as different things. If they asked about the patients’ sexuality, they were asked how they did this and how often. It was specified in the questionnaire that this was related to outpatient interviews in order to guarantee that the interviews would occur in private office settings despite it being a teaching hospital. This condition was stated in a message at the beginning of the survey.

If sexuality was addressed, the participants were asked if they were embarrassed by the same sex and/or opposite sex patients, if they found diverse sexual orientations in elderly patients, and if they found any relation with a specific type of pain. For those who did not ask about sexuality, they were asked the reasons, if they thought that seniors are sexual beings, and if chronic pain was related to sexual dysfunctions.

Statistical analysis used Statistical Package for the Social Sciences (SPSS), version 17.0, Minitab 16, and Microsoft Excel 2010 software. Analysis of variance (ANOVA) was used for the comparison of means, and the *χ* ^*2*^ test to check the statistical significance of the two variables. The confidence interval of the statistically significant relations was 95% with a p value ≤0.05.

## RESULTS

The characteristics of the 155 physicians are outlined in table 1.

**Table 1:** Sample characteristics

	n (%)
Specialty	
Acupuncture	5 (3.2)
General Practice/Internal Medicine	59 (38.1)
General Surgery	1 (0.6)
Endocrinology	12 (7.7)
Physical Medicine and Rehabilitation	7 (4.5)
Geriatrics	37 (23.9)
Infectious Diseases	4 (2.6)
Sports Medicine	1 (0.6)
Neurology	10 (6.5)
Orthopedics	7 (4.5)
Rheumatology	12 (7.7)
Patient age (years)	
60-69	71 (44.9)
70-79	77 (48.7)
≥80	10 (6.3)
Type of pain	
Nociceptive	57 (34.3)
Neuropathic	19 (11.4)
Dysfunctional	44 (26.5)
Psychogenic	5 (3.0)
Mixed	41 (24.7)

The physicians reported attending patients mostly between 70-79 years. The most frequent type of pain reported by the physicians was nociceptive. Most physicians reported not addressing the issue of sexuality. Fifty-six physicians did address the issue, accounting for 36.1% of sample.

More experienced physicians addressed sexuality significantly more frequently compared to less experienced physicians. Among those who addressed the issue of sexuality, the mean years of experience was 6.09, as compared to 3.8 in the non-addressing group, with a p value of 0.009.

Receiving training in sexual history taking had a statistically significant relation with addressing the issue of sexuality. Among those who addressed the issue, 29.63% had received training, while among the non-addressing group, 21.27% had received training. An absolute change of 8.36% represents the number needed to treat, which was 12.

Among those who addressed the patients’ sexuality, 22% reported doing it in more than 60% of interviews. Most did it in less than 30% of interviews. Most (72.9%) of these physicians did not report any embarrassment in taking the sexual history; 74.2% of physicians understand sex and sexuality as different issues, while 17.4% of physicians think they are different topics, but that the difference is irrelevant for clinical practice (Table 2).

**Table 2:** Addressing sexuality by physicians

	n (%)	p value
Addressing sexuality		
Yes	56 (36.1)	<0.001
No	99 (63.9)	
Frequency of addressing sexuality in the appointments		
<30%	25 (42.4)	
31-60%	21 (35.6)	0.450
>60%	13 (22.0)	0.018
Embarrassment in addressing the issue of sexuality		
Yes	16 (27.1)	<0.001
No	43 (72.9)	
Sex and sexuality		
Different	115 (74.2)	
The same	6 (3.9)	<0.001
Difference is not relevant	27 (17.4)	<0.001
Do not know/No answer	7 (4.5)	<0.001
Diverse sexual orientations		
Did not perceive	38 (66.7)	
More common in men	10 (17.5)	<0.001
More common in women	0	<0.001
No predominance	9 (15.8)	<0.001
Sexually active elderly are normal		
Yes	86 (86.9)	
No	3 (3.0)	<0.001
No answer	10 (10.1)	<0.001
Sexual history taking training		
Received	36 (23.2)	<0.001
Did not receive	112 (72.3)	
No answer	7 (4.5)	<0.001

Most participants did not perceive any relation between sexual problems and a specific type of chronic pain. Roughly 87% of physicians who did not address sexuality found it normal for older individuals to have a sexual life. The vast majority of the sample did not receive any training in sexual history taking (Table 2).

**Figure 1:**
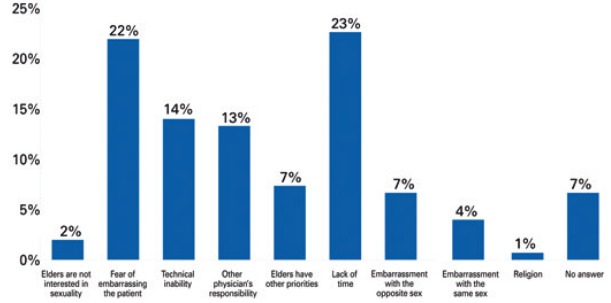
Reasons for not addressing sexuality

Most physicians who addressed sexuality did not notice a clear relationship with a specific type of chronic pain. More than half of physicians (51.5%) who did not address the issue of sexuality reported chronic pain as potentially being related to sexual dysfunction; and 86.9% of those would be willing to change their practice patterns if it were proven that it had an impact on the patients’ quality of life. Among 99 physicians who did not take sexual history, 22.8% did not do it due to lack of time, 22.1% justified it by fear of embarrassing the patient, and 14.1% felt unable to do it. About 7% did not answer and 13.1% felt it was another physician’s responsibility (Figure 1).

Geriatrics and Endocrinology were the specialties that addressed the issue of sexuality more often, with no statistically significant difference between them. Addressing sexuality was much less frequent among other specialties (Table 3).

**Table 3:** Addressing sexuality according to specialty

Specialty	No address n (%)	Address n (%)	p value
General Practice/Internal Medicine	48 (83)	10 (17)	<0.01
Endocrinology	3 (25)	9 (75)	0.126
Physical Medicine and Rehabilitation	6 (86)	1 (14)	<0.01
Geriatrics	7 (19)	30 (81)	
Neurology	8 (80)	2 (20)	<0.01
Orthopedics	7 (100)	0	<0.01
Rheumatology	10 (83)	2 (17)	<0.01

Orthopedic surgeons did not take sexual history and geriatricians did it more often. Neurologists and rheumatologists communicated a lack of time as the main reason for not addressing the issue of sexuality. Physical Medicine and Rehabilitation Specialists feared embarrassing the patient. Geriatricians mentioned technical inability, and orthopedic surgeons believed another physician was responsible for the issue (Table 4).

**Table 4:** Frequency of reasons for not addressing the issue of sexuality according to specialty

Reasons for not addressing sexuality	General Practitioner Internal Medicine	Physical Medicine and Rehabilitation	Geriatrics	Neurology	Orthopedics	Rheumatology	Total

	n (%)	n (%)	n (%)	n (%)	n (%)	n (%)	n (%)
Fear of embarrassment	18 (26)	3 (30)	2 (22)	1 (10)	2 (25)	3 (25)	29 (24)
Inability	10 (14)	1 (10)	4 (44)	2 (20)	0 (0)	1 (8)	18 (15)
Responsibility of other physicians	8 (11)	2 (20)	0 (0)	2 (20)	6 (75)	0	18 (15)
Others priorities	8 (11)	0	1 (11)	2 (20)	0	0	11 (9)
Lack of time	19 (27)	2 (20)	2 (22)	3 (30)	0	8 (67)	34 (29)
Embarrassed by the opposite sex	7 (10)	2 (20)	0	0	0	0	9 (8)

Total	70 (59)	10 (8)	9 (8)	10 (8)	8 (7)	12 (10)	119 (100)

p=0.002.

Most participants reported questioning the patient directly, rather than waiting for the patient to ask, or asking subtle questions, regardless of the patient’s gender. There was a slight decrease in the rate of asking those questions directly to the patient of the opposite sex. Three physicians reported using indirect addressing of the issue, such as asking about marital status and relationship status.

## DISCUSSION

Sexuality still is, especially when related to elders, a taboo. Except for a few specialties that naturally address the issue of sexuality, such as Gynecology and Urology, most physicians still ignore/neglect the matter.

In the present study, the sample included physicians from eleven diverse specialties. The mean physician experience was relatively low. However, this could be justified by the study setting, at a teaching hospital, which includes several newly graduated physicians in their residency programs (although they were not considered separate). That is not necessarily a negative bias, since it could give a truer picture of medical schools in Brazil.

It is very difficult to evaluate the content of medical appointments through interviews. The physician could be inhibited for being evaluated by a colleague, or not allow his/her patient to be assessed after the appointment. Patients, on the other hand, could be embarrassed to discuss such intimate matters with an unknown physician. In order to overcome these barriers, an anonymous self-administered questionnaire was chosen for use in this study.

Coexistence of chronic pain can negatively impact sexual function,^(19)^ which already has barriers related to senescence itself.^(12)^ In this study, addressing the issue of sexuality for elderly patients with chronic pain was broader than it was in the existing literature. Here approximately one-third of participants referred to it. This could be explained again by the study being conducted in a teaching hospital setting where a thorough history-taking technique is regularly taught. The proximity between specialties can also be a reason since this promotes the exchange of knowledge.

Geriatrics is a specialty with the highest proportion of addressing the issue of sexuality, probably because it studies the impact of aging in the human body and understanding complications of multimorbidity. The Comprehensive Geriatric Assessment is usually a longer interview that allows for more intimate matters to surface easier.

Endocrinology, possibly due to the study of diseases with a high impact on sexual functions (hormonal dysfunctions, diabetes), had a rate of addressing comparable to geriatrics. Orthopedic surgeons addressed the issue the least. They justified this by reporting that the topic is not related to their specialty. Nevertheless, many elders have an orthopedic surgeon as their only physician, or their primary attending physician, when they complain of chronic pain.

The main reason reported for not addressing the issue of sexuality in this study was a lack of time during interview. This could be a reflection of the Brazilian public health system, in which time is very reduced, worsening the quality of appointment.^(25)^ This setting makes it difficult for matters of “lesser” importance to physicians and patients (*e.g.*, sexuality) to show. It is necessary to rethink this style of medicine that gives priority to more interviews per hour rather than a comprehensive evaluation. There should be triage strategies for sexual dysfunction in shorter interviews.

Another allegedly important reason for not addressing the issue of sexuality was the feeling of technical inability. Although sex Medicine is widely neglected in the syllabus of most medical schools, this absence of expertise is not a valid reason. A triage could be performed and a referral to a specialized colleague could be offered to the patient.

Among the physicians who addressed sexuality in their appointments, only 13 discussed the issue in most events, which accounts for 8.38% of sample. This is very unsatisfying, since the prevalence of sexual dysfunction in chronic pain patients could be as high as 73%.^(19)^ Treating these individuals can bring benefits to quality of life and pain control because good sexual health is related to better pain control.^(10)^

There was a statistically significant difference in the number of years of medical experience between the addressing and non-addressing groups in this study. This result could be associated to two different reasons: First: more experience could mean greater maturity concerning sexual issues, and/or Second: changes made in the medical undergraduate course syllabus regarding approach of patient’s sexuality more difficult for younger physicians.

Being trained to take sexual history during the undergraduate course had a statistically significant and positive effect in the rate of positive responses to addressing the issue of sexuality. While in absolute numbers there was a slight change (an increase of 8.36%), this represents a number needed to treat of 12. This means that it takes 12 medical students to be trained in order to have one of them addressing sexuality of their patients routinely. This is a very good result considering the low cost involved. According to Lindau et al.,^(26)^ two medical undergraduate student cohorts were assessed on their ability to take a sexual history; one had been trained in this matter and the training had a much better result for those cases in which the dysfunction was obvious. However, for subtle cases, it failed to show a difference from no training at all. Another study^(27)^ offered various strategies for teaching sexual history taking in medical schools.

Regarding the difference between sex and sexuality, most of the sample knew there was a difference, irrespective of whether sexuality was addressed or not. It is worth mentioning that some physicians knew there was a difference, but thought it had no impact on clinical practice. Sex relates to actual intercourse. It is during sex that most dysfunctions appear. Sexuality is a broader concept, regarding the projection of sex in the patient’s “self,” including their expectations, frustrations, and series of actions related to sex itself, such as flirting, sexual fantasies, caressing, and masturbation.

Most participants did not report encountering sexual minorities in their practice. This does not correspond to reality according to the current Brazilian literature.^(28)^ Perhaps it is a reflection of addressing the issue related to sex instead of sexuality. Additionally, it could be related to difficulties experienced by the elderly to come out/show their true sexuality.^(23)^ Since sexuality of the aged is a taboo, a greater problem can be expected regarding sexual minorities.

Because of cultural nuances in Brazil, where elderly people are considered asexual beings, older adults with sexual dysfunctions might be inhibited to start a conversation about their sexuality. This demands a proactive role from the physician. A study conducted in the same environment as this one showed that 68.8% of very old women with chronic pain would like the physician to address the matter.^(29)^

Many patients seek medical care for treating chronic pain, ignoring the fact that possible concomitant sexual dysfunction can be treated as well. The patient is not expected to know that both problems could be related. The attending physician should be aware that there is a relation between them and treating the pain while ignoring sexual function can further worsen the sexuality issue.

## CONCLUSION

The present study showed that most physicians fail to address the issue of the sexuality of their elderly patients with chronic pain. It was also observed there were significant differences in the rate of addressing the issue among the medical specialties, with geriatrics and endocrinology performing it more often. The main reasons for not doing so were lack of time, fear of embarrassing the patient, and feelings of technical inaptitude. Longer experience in clinical practice was associated to a higher rate of addressing the issue of sexuality. It is necessary to develop strategies to improve instances of addressing the issue for elderly with chronic pain, by improving training of undergraduate medical students and performing continued medical education, in addition to promoting a positive impact on the quality of life of older adults.

## References

[1] Instituto Brasileiro de Geografia e Estatística (IBGE) (2013). Projeção da população por sexo e idade: 2000-2060. Projeção da população das unidades federativas por sexo e idade: 2000-2030.

[2] Lindau ST, Schumm LP, Laumann EO, Levinson W, O’Muircheartaigh CA, Waite LJ (2007). A study of sexuality and health among older adults in the United States. N Engl J Med.

[3] Bretschneider JG, McCoy NL (1988). Sexual interest and behavior in healthy 80- to 102-year-olds. Arch Sex Behav.

[4] Kalra G, Subramanyam A, Pinto C (2011). Sexuality: desire, activity and intimacy in the elderly. Indian J Psychiatry.

[5] Helgason AR, Adolfsson J, Dickman P, Arver S, Fredrikson M, Göthberg M (1996). Sexual desire, erection, orgasm and ejaculatory functions and their importance to elderly Swedish men: a population-based study. Age Ageing.

[6] Griffiths E (1988). No sex, please, we’re over 60. Nurs Times.

[7] Drench ME, Losee RH (1996). Sexuality and sexual capacities of elderly people. Rehabil Nurs.

[8] Bytheway B (1995). Ageism. Get your knickers off granny: interpersonal relations.

[9] Chen X, Zhang Q, Tan X (2009). Cardiovascular effects of sexual activity. Indian J Med Res.

[10] Brody S (2010). The relative health benefits of different sexual activities. J Sex Med.

[11] Onder G, Penninx BW, Guralnik JM, Jones H, Fried LP, Pahor M (2003). Sexual satisfaction and risk of disability in older women. J Clin Psychiatry.

[12] Masters WH, Johnson VE (2010). Human sexual response.

[13] Paice J (2003). Sexuality and chronic pain. Am J Nurs.

[14] Kessel B (2001). Sexuality in the older person. Age ageing.

[15] Patel NB, Kopf A, Patel NB (2010). Physiology of pain. Guide to pain management in low-resource settings.

[16] Brattberg G, Parker MG, Thorslundb M (1996). The prevalence of pain among the oldest old in Sweden. Pain.

[17] Roy R, Thomas M (1986). A survey of chronic pain in an elderly population. Can Fam Physician.

[18] Dellaroza MS, Pimenta CA, Matsuo T (2007). Prevalência e caracterização da dor crônica em idosos não institucionalizados. Cad Saude Publica.

[19] Ambler N, Williams AC, Hill P, Gunary R, Cratchley G (2001). Sexual difficulties of chronic pain patients. Clin J Pain.

[20] Bahouq H, Fadoua A, Hanan R, Ihsane H, Najia HH (2013). Profile of sexuality in Moroccan chronic low back pain patients. BMC Musculoskelet Disord.

[21] Gott M, Hinchliff S (2003). Barriers to seeking treatment for sexual problems in primary care: a qualitative study with older people. Fam Pract.

[22] Moreira ED, Brock G, Glasser DB, Nicolosi A, Laumann EO, Paik A, Wang T, Gingell C, GSSAB Investigators’ Group (2005). Help-seeking behaviour for sexual problems: the global study of sexual attitudes and behaviors. Int J Clin Pract.

[23] Lindau ST, Halter JB, Ouslander JG, Tinetti ME, Studenski S, High KP, Asthana S (2009). Sexuality, sexual function, and the aging woman. Hazzard’s geriatric medicine and gerontology.

[24] Miot HA (2011). Tamanho da amostra em estudos clínicos e experimentais. J Vasc Bras.

[25] Caprara A, Rodrigues J (2004). A relação assimétrica médico-paciente: repensando o vínculo terapêutico. Cien Saude Colet.

[26] Lindau ST, Goodrich KG, Leitsch SA, Cook S (2008). Sex in the curriculum: the effect of a multi-modal sexual history-taking module on medical student skills. Sex Educ Sex Soc Learn.

[27] Skelton JR, Matthews PM (2001). Teaching sexual history taking to health care professionals in primary care. Med Educ.

[28] Mota MP (2009). Homossexualidade e envelhecimento: algumas reflexões no campo da experiência. SINAIS - Rev Eletrôn.

[29] Santos AM, Santos FC, Cendoroglo MS (2015). Sexualidade e dor crônica: sexualidade em idosas longevas com dor crônica e a descrição de fatores de interferência. Rev Dor.

